# Tension–Temperature Synergy in Tailoring Surface Polarity and Interfacial Properties of High-Modulus PAN-Based Carbon Fibers

**DOI:** 10.3390/ma19163514

**Published:** 2026-08-19

**Authors:** Aijun Gao, Tiansheng Fan, Weize Tian, Panpan Xu, Hailong Zhang

**Affiliations:** 1 College of Materials Science and Engineering, Beijing University of Chemical Technology, Beijing 100029, China; 2025210240@buct.edu.cn (T.F.); tianwz.bjhy@sinopec.com (W.T.); 2 Jiangsu Hengshen Co., Ltd., Danyang 212300, China; sqxupanpan@163.com; 3 School of Materials Science and Engineering, North China University of Water Resources and Electric Power, Zhengzhou 450045, China

**Keywords:** high-modulus carbon fiber, tension–temperature synergy, surface polarity, interfacial shear strength, graphitization

## Abstract

**Highlights:**

- Temperature and tension exert decoupled control over crystallite size and axial orientation in high-modulus carbon fibers.
- A competitive balance keeps surface polarity nearly unchanged under varying tension at fixed temperature.
- Low-temperature high-tension processing achieves ~350 GPa modulus with 37.9% higher interfacial shear strength.
- The Sac parameter shows strong linear correlations with surface energy and interfacial shear strength.

**Abstract:**

High-temperature graphitization inevitably compromises the surface polarity and resin wettability of polyacrylonitrile (PAN)-based high-modulus carbon fibers (HMCFs), creating a long-standing trade-off between fiber modulus and interfacial adhesion that restricts its applications. Here we report a tension–temperature synergy to overcome this limitation. HMCFs were fabricated at 1700–2100 K under axial tensions of 0–70 N, and the resulting microstructures and surface activity were characterized by X-ray diffraction, Raman spectroscopy, dynamic contact angle testing, and microdroplet debond measurements. Temperature dominates crystallite coarsening and surface-active carbon (Sac) concentration, whereas tension enhances axial lamellar orientation without inducing appreciable grain growth. At constant temperature, two competing effects, both slight crystallite growth and radial lamella rearrangement, keep Sac stable under varying tension. Fibers processed at 1900 K with 60 N tension achieve a modulus of ~350 GPa, equivalent to that of the 2100 K/10 N sample, while delivering a 13.5% higher Sac, elevated surface energy (26.3 mN·m^−1^), and 37.9% stronger interfacial shear strength (IFSS). The Sac parameter exhibits strong correlations with surface energy and IFSS. This one-step in situ thermal strategy eliminates post-treatment and offers an industrially viable route to HMCFs with balanced modulus and intrinsic interfacial bonding.

## 1. Introduction

Polyacrylonitrile (PAN)-derived high-modulus carbon fibers (HMCFs, tensile modulus > 350 GPa) are indispensable lightweight reinforcements for aerospace primary load-bearing structures, owing to their exceptional specific modulus and dimensional stability at elevated temperatures [1,2,3]. However, conventional graphitization at temperatures exceeding 2000 K significantly enhances graphite crystallinity and crystallite dimensions, which inevitably eliminates edge-active carbon atoms on the fiber surface [4]. This surface degradation leads to low polarity, poor wettability by resin matrices, and consequently weak fiber–matrix interfacial shear strength (IFSS) [5]. Relative to medium-modulus carbon fibers (~290 GPa), HMCFs typically exhibit a 30–40% reduction in interfacial performance, which severely constrains their application in composite structures subjected to complex alternating loads [2].

Conventional approaches to improving carbon fiber interfacial properties rely predominantly on post-surface treatments, including anodic oxidation [6,7,8], plasma etching [9,10,11,12], and chemical grafting [13,14,15,16]. Nevertheless, the effectiveness of these secondary modifications is fundamentally constrained by the intrinsic graphitic structure of the as-produced fibers. Highly graphitized HMCFs possess few surface-active sites, establishing an inherent upper limit for interfacial enhancement regardless of post-treatment parameters [5,17,18]. This limitation underscores the need for an in situ thermal processing strategy that can generate polar surface structures during graphitization itself, without compromising the fiber’s bulk mechanical properties.

Tension applied during continuous high-temperature graphitization represents a critical adjustable parameter for tailoring carbon fiber microstructures [19]. Adequate axial tension restricts thermal shrinkage of the fiber and promotes alignment of graphite lamellae along the fiber axis, which effectively elevates tensile modulus without inducing excessive growth of graphite crystallites [20]. Previous studies have established that increased tension substantially improves microcrystalline orientation, whereas high graphitization temperatures simultaneously accelerate crystallite growth and diminish surface-active carbon content [21]. However, most earlier investigations examined the isolated effects of temperature or tension separately, with limited attention to the interactive influence of graphitization temperature and drawing tension on both the bulk graphite structure and intrinsic surface polarity [22,23]. Moreover, quantitative indicators for evaluating the surface polarity of HMCFs remain scarce, resulting in an ambiguous correlation among processing parameters, microstructures, and interfacial performance.

In this paper, HMCFs samples were prepared under gradient tension (0–70 N) at various graphitization temperatures (1700 K, 1900 K and 2100 K). X-ray diffraction (XRD), Raman spectroscopy, dynamic contact angle measurements, and microdroplet debond tests were employed to systematically elucidate the distinct roles of tension in modulating crystallite size, radial orientation, and surface-active carbon (Sac) content. A competitive mechanism between microcrystal coarsening and radial graphitic lamella exposure induced by axial tension is proposed to rationalize the nearly invariant surface polarity observed under variable tension. Furthermore, a tension–temperature synergistic regulation route is proposed. Substantial drawing tension combined with moderate graphitization temperature achieves comparable fiber modulus while preserving abundant edge-active carbon sites. This work offers a novel processing paradigm for fabricating HMCFs with superior intrinsic interfacial activity.

## 2. Materials and Methods

### 2.1. Materials

Polyacrylonitrile (PAN) precursor fibers (0.78 dtex, linear density 0.940 g/m) were adopted as the starting fiber raw material, prepared by dry–wet spinning at the National Carbon Fiber Engineering Research Center (Beijing, China). E51 epoxy resin (bisphenol-A diglycidyl ether type), methyltetrahydrophthalic anhydride (MTHPA) curing agent, 2,4,6-tris(dimethylaminomethyl) phenol (DMP-30) accelerator were purchased from Sinopharm Chemical Reagent Co., Ltd. (Shanghai, China). All chemical reagents were used as received without further purification.

### 2.2. Preparation of HMCFs

A continuous multi-stage thermal treatment line was employed to produce the HMCFs. The production line consisted of an air stabilization furnace, a low-temperature carbonization furnace, and a high-temperature graphitization furnace, with high-purity nitrogen (99.999%) serving as the protective atmosphere throughout the entire heat treatment process.

Stabilization stage: The precursor tows were subjected to gradient heating temperatures ranging from 483 to 523 K under constant low tension to avoid molecular chain disorientation and to ensure uniform thermal oxidative stabilization across the filament cross-section.

Low-temperature carbonization: The stabilized fibers were passed through a furnace at temperatures ranging from 623 to 953 K to eliminate most of the hydrogen, oxygen, and partial nitrogen heteroatoms, thereby converting the stabilized PAN structure into a turbostratic carbon framework.

**Figure 1:**
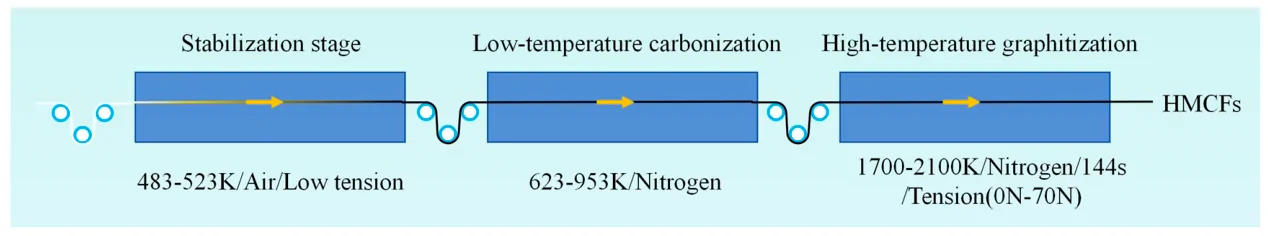
Schematic diagram of HMCFs preparation.

High-temperature graphitization: The residence time was fixed at 144 s for all experimental groups, and two independent variables were systematically regulated: graphitization temperature (1700 K, 1900 K, and 2100 K) and axial drawing tension (ranging from 0 N to 70 N). The fiber tension was precisely monitored and adjusted via a SCHMIDT DN1-5000 online tension tester installed at the outlet of the graphitization furnace. The tension gradient groups were set at approximately 10 N, 20 N, 30 N, 40 N, 50 N, 60 N, and 70 N, with a free-shrinkage control group (0 N). After stable temperature and tension equilibrium were achieved, the finished fiber tows were wound and collected for subsequent characterization. Figure 1 illustrates the preparation process. Table S1 lists the physical properties of HMCFs at different tension and temperature.

### 2.3. Characterizations

#### 2.3.1. X-Ray Diffraction

XRD patterns were recorded on an X’Pert PRO MPD diffractometer (Malvern Panalytical, Almelo, Overijssel, The Netherlands) equipped with Cu Kα radiation (λ = 0.154 nm). Equatorial scanning over the 10–90° range with a step width of 0.01313° was used to determine the interlayer spacing d_002_ from the (002) peak position via Bragg’s law (Equation (1)), and the apparent crystallite size perpendicular to the basal plane (Lc) from the (002) peak width via the Scherrer equation (Equation (2)). Meridional scans were collected for the (100) diffraction peak to determine the apparent crystallite size parallel to the basal plane (La) using the Scherrer equation (Equation (2)). Azimuthal scans from 90° to 270° with a step size of 0.5° at the (002) peak position were employed to evaluate the graphite microcrystalline orientation factor (π) via Equation (3).

(1) 2dsinθ=nλ

(2) $$L=\frac{K\lambda }{\beta \cos\theta }$$

(3) $$\pi =\frac{180-2Z}{180}\times 100\%$$

while d is the interlayer spacing; θ is the diffraction angle; n is the diffraction order; λ is the X-ray wavelength (0.154 nm); L is the average microcrystal size perpendicular to the crystal plane; K is the shape factors (0.9 for Lc, 1.84 for La); π is the orientation degree; 2Z is the half-width at half maximum of the azimuthal intensity distribution, which quantifies the degree of preferred alignment of graphite basal planes along the fiber axis. All peak deconvolution and parameter extraction were performed using the instrument’s accompanying software package. The equatorial, meridional, and azimuthal XRD patterns are presented in Figures S1–S3. For each sample condition, XRD patterns were acquired from a single representative specimen, consistent with standard practice in carbon fiber structural characterization.

#### 2.3.2. Raman Spectroscopy

Raman spectra were acquired using an inVia RM2000 micro-Raman spectrometer (Renishaw plc, Wotton-under-Edge, Gloucestershire, UK) with a 532 nm excitation laser to probe the carbon chemical structure and to establish the surface-active carbon index (Sac). Three sampling methods were designed to distinguish surface cross-section radial skin-core structure. For surface analysis, single filaments were mounted flat onto glass slides and measured with a 50× objective, using 10% laser power over the 1000–1900 cm^−1^ spectral range with an acquisition time of 15 s and five accumulations per specimen. For cross-sectional radial profiling, fibers were transversely sectioned to expose the full circular face, and spectra from the skin and core regions were collected separately under a 100× objective.

The Sac indicator, reflecting the relative proportion of edge-active carbon atoms exposed on the fiber surface, was defined as:

(4) $$Sac=\frac{I_{D}}{I_{D}+I_{G}}$$

where *I_D_* and *I_G_* are the integrated areas of the D band (~1350 cm^−1^, associated with defects and edge carbons) and the G band (~1580 cm^−1^, corresponding to in-plane sp^2^ graphitic carbons), respectively.

For comparison, the conventional graphitization index *R* = *I_D_*/*I_G_* was also calculated for each specimen. All spectral data were baseline-corrected and normalized prior to peak fitting using a mixed Gaussian–Lorentzian function.

#### 2.3.3. Dynamic Contact Angle and Surface Energy Calculation

**Figure 2:**
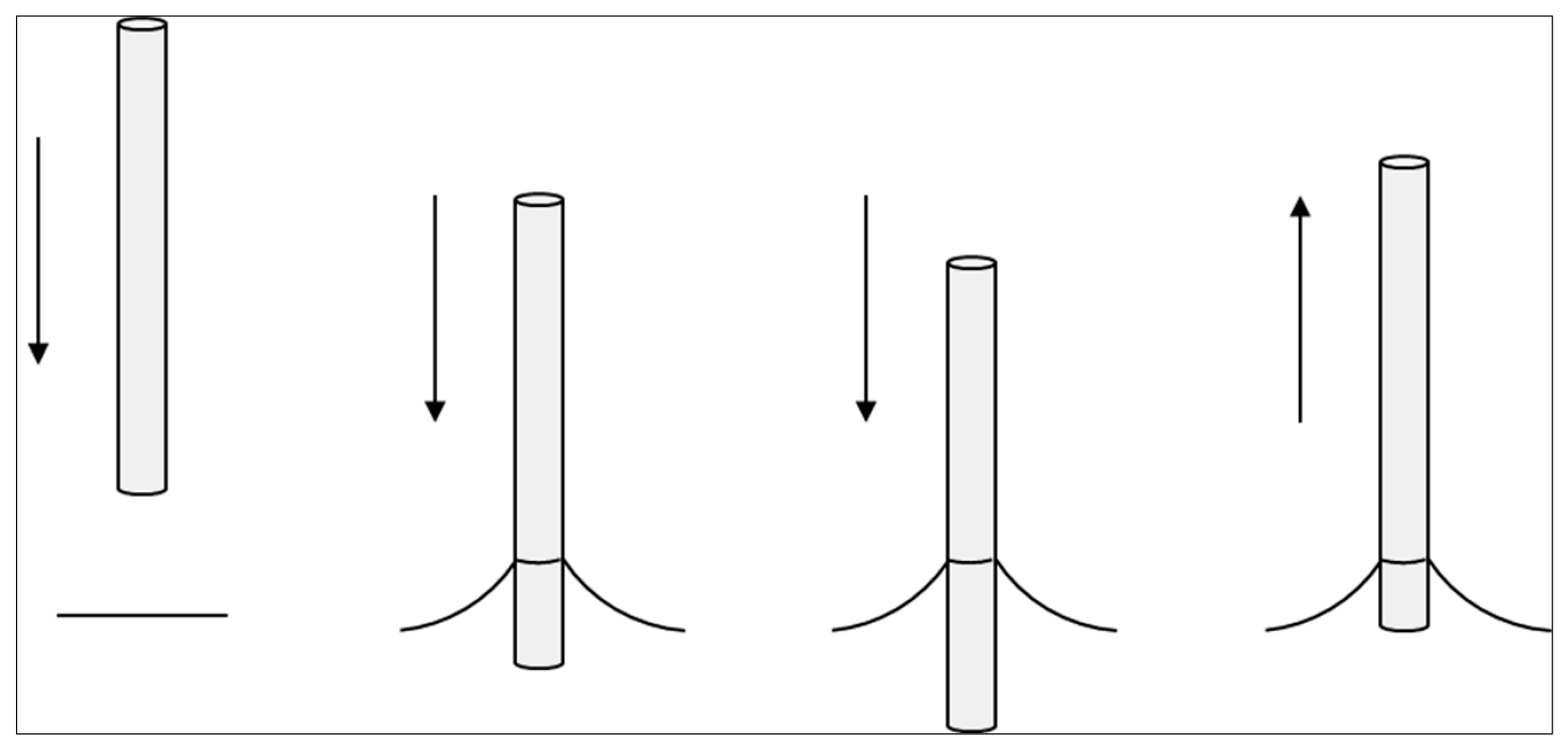
Schematic diagram of dynamic contact angle test [2].

Dynamic contact angles were measured using a DCAT21 tensiometer (Dataphysics Instruments GmbH, Filderstadt, Baden-Württemberg, Germany) following the Wilhelmy plate method. Four monofilaments of 8 mm length were mounted in parallel on the sample holder. The immersion speed was set to 0.08 mm/s, the immersion depth to 5 mm, and the force threshold to 0.08 mg. Three consecutive measurement cycles were performed for each test liquid, and the average advancing angle test is shown in Figure 2.

Surface energy of the fibers was calculated using the Owens–Wendt–Rabel–Kaelble (OWRK) method. The OWRK equation, which separates the surface energy into dispersive and polar components, is expressed as:

(5) $$\gamma_{L}(1+\cos\theta )\;={2(\gamma }_{L}^{p}\gamma_{s}^{p}{)}^{1/2}+{2(\gamma }_{L}^{d}\gamma_{s}^{d}{)}^{1/2}$$

(6) $$\gamma_{s}=\gamma_{s}^{p}+\gamma_{s}^{d}$$

where $\gamma_{s}$ is the total surface energy of the fiber ($\gamma_{s}=\gamma_{s}^{d}+\gamma_{s}^{p}$); $\gamma_{L}$ is total surface energy of the liquid; $\gamma_{s}^{d}$ and $\gamma_{s}^{p}$ is the dispersive and polar components of the fiber surface energy; $\gamma_{L}^{d}$ and $\gamma_{L}^{p}$ is the dispersive and polar components of the liquid surface tension; θ is advancing contact angle. The properties of the two testing liquids used in this study are summarized in Table S2.

#### 2.3.4. Single-Fiber Microdroplet Debond Test

The IFSS between the carbon fiber and the epoxy matrix was assessed through the single-fiber microdroplet debond test, a widely accepted method for characterizing fiber–matrix adhesion. The test followed an established laboratory protocol based on the microdroplet debond method [2], consistent with procedures commonly reported in the carbon fiber literature [3,15]. The epoxy matrix was prepared from E51 epoxy resin, with MTHPA as the curing agent and DMP-30 as the accelerator, at a weight ratio of EP:MTHPA:DMP-30 = 100:85:1.

**Figure 3:**
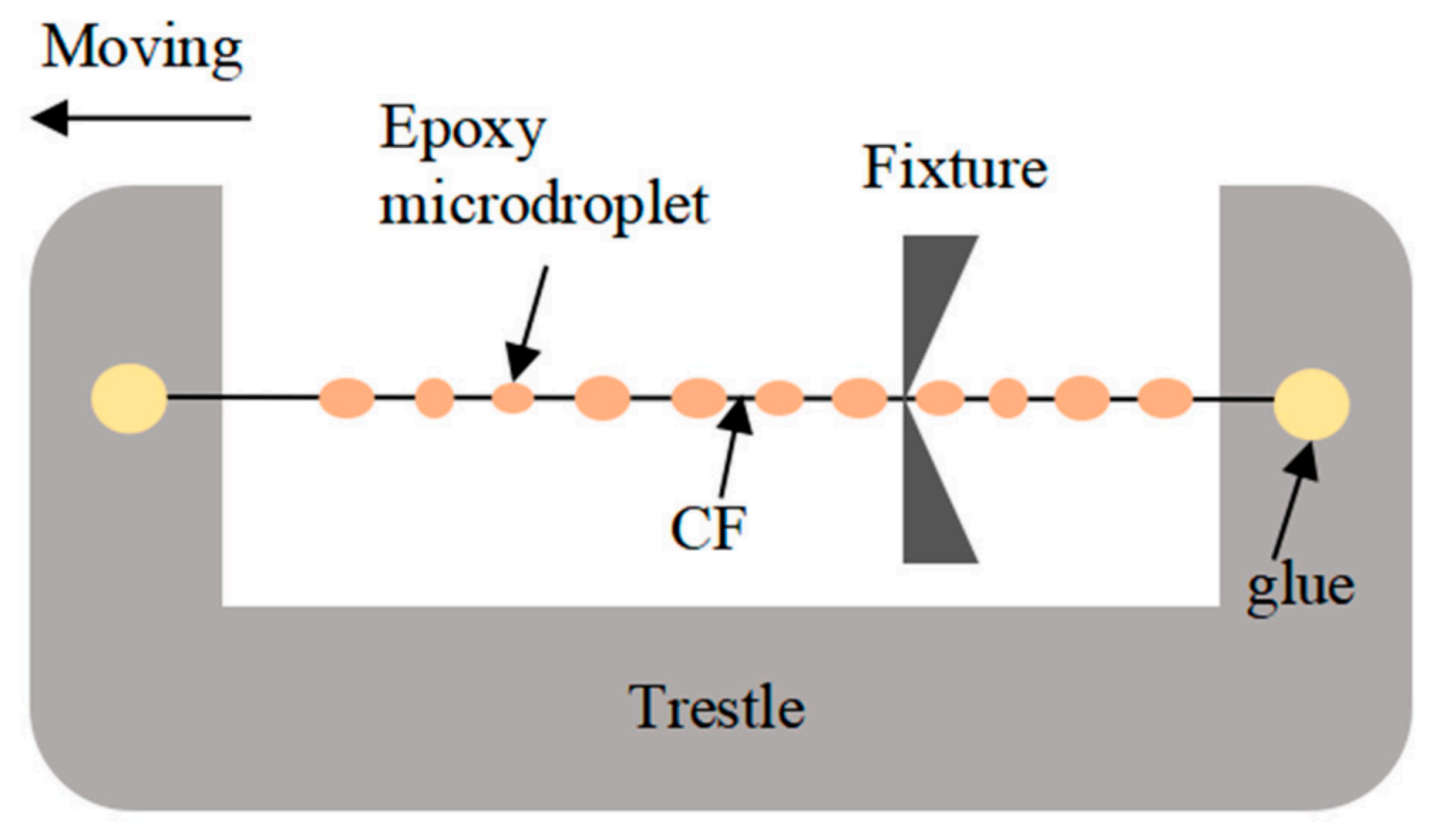
Schematic diagram of microdroplet debond test [2].

Single filaments were mounted on custom-designed metal frames, and small epoxy droplets with an embedded length of 40–60 μm were deposited onto the fiber surface, followed by curing at 120 °C for 3 h. During testing, the resin droplet was clamped by two blades while the fiber was pulled at a constant displacement rate of 0.1 mm/min, as shown in Figure 3. Identical testing parameters, including displacement rate, droplet curing conditions, blade gap settings, and data acquisition criteria, were applied consistently across all fiber groups to ensure the comparability of IFSS measurements.

The IFSS value was calculated using Equation (7):

(7) $$\mathrm{IFSS}=\frac{F_{\max}}{\pi dL}$$

where *F*_max_ is the maximum debonding force; d is the fiber diameter; L is the embedded length of the droplet. For each fiber group, fifteen valid droplets were tested to minimize statistical scattering.

#### 2.3.5. Tensile Mechanical Properties

Tensile modulus of the fiber tows was determined using an Instron 5967 universal testing machine (Instron Corporation, Norwood, MA, USA), in accordance with GB/T 3362-2017 (Test methods for tensile properties of carbon fiber multifilament) [24]. This standard is applicable to the determination of tensile strength, tensile modulus of elasticity, and elongation at break for 1 K–24 K carbon fiber multifilaments after impregnation and curing. In our work, the fiber tow (12 K) was impregnated with epoxy resin and cured, following the guidelines of GB/T 3362-2017, to prevent filament sliding and ensure uniform load transfer during testing.

Specimens of 200 mm in length were prepared by impregnating the tows with epoxy resin, followed by curing. Reinforced end tabs were attached at both ends to prevent premature failure at the grips. The crosshead speed was set at 2 mm/min, and an extensometer was used for accurate strain measurement during modulus determination. For each sample group, ten replicate specimens were tested. The tensile modulus was calculated from the linear slope of the stress–strain curve in the elastic deformation region, and the measured modulus values were normalized by linear density and bulk density before averaging.

## 3. Results and Discussion

### 3.1. Modulus Evolution Under Different Tension–Temperature Combinations

**Figure 4:**
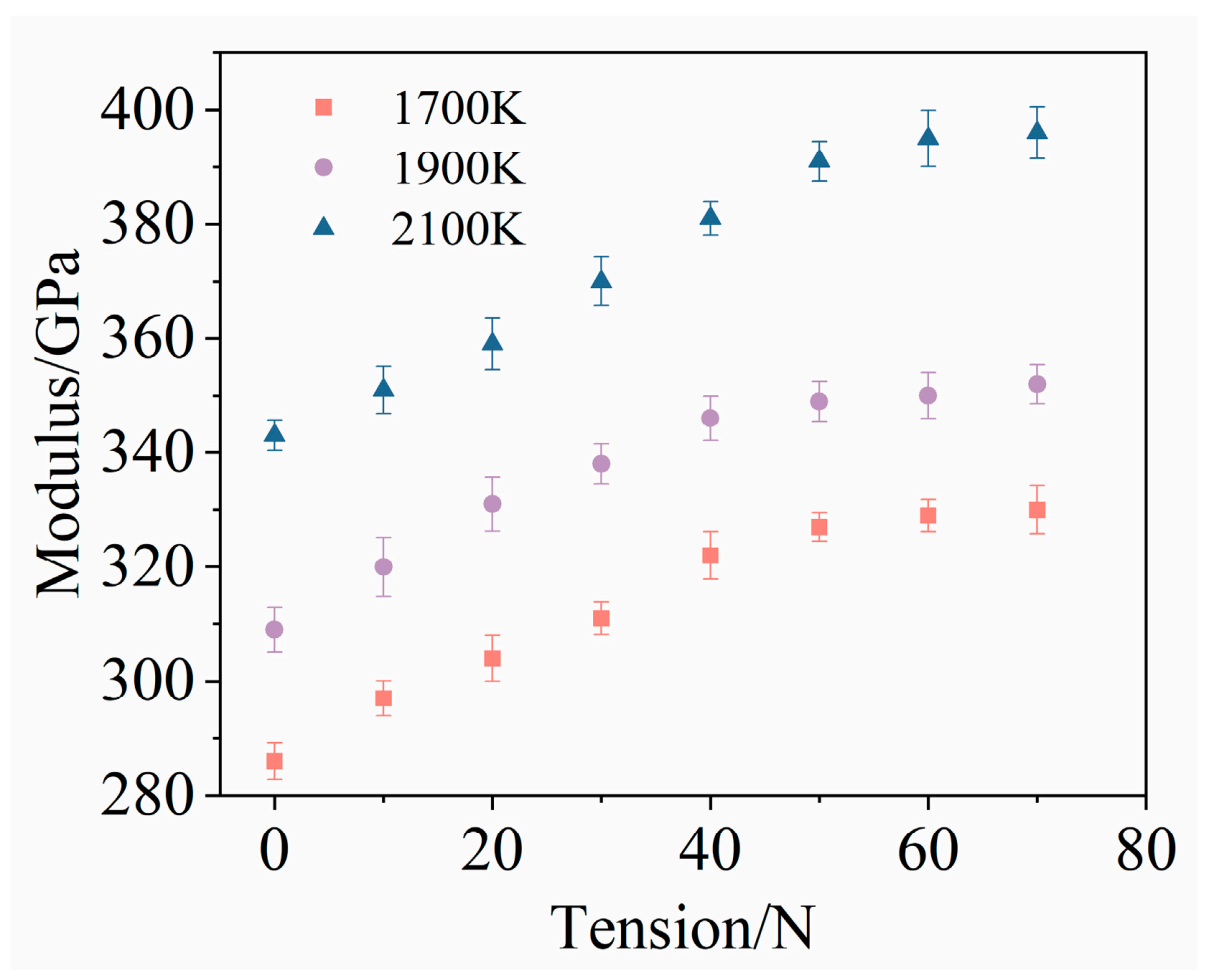
Tensile modulus of carbon fibers at different tension–temperature.

Figure 4 shows the tensile modulus of PAN-based carbon fibers as a function of axial tension at different graphitization temperatures (1700 K, 1900 K and 2100 K). At every fixed temperature, fiber modulus increases steadily as the applied axial tension increases from 0 N to 70 N. Regardless of tension level, fibers treated at higher graphitization temperature consistently yield higher modulus values, confirming that both thermal energy and axial tensile load contribute synergistically to improving stiffness [25].

For specimens graphitized at 2100 K, the modulus starts at 343 GPa under zero tension and gradually climbs to nearly 396 GPa at the maximum tension. The rate of modulus growth diminishes when tension exceeds 50 N, suggesting that axial alignment of graphite lamellae approaches saturation under high tension, and further increasing tensile load cannot substantially improve the preferred orientation of crystallites along the fiber axis [26]. The same trend is observed for 1700 K, 1900 K and 2100 K groups.

A comparison of the modulus increments reveals that both graphitization temperature and axial tension contribute significantly to modulus enhancement within the experimental range. Raising tension from 0 N to 70 N at 1900 K increases the modulus by approximately 43 GPa, while elevating the temperature from 1900 K to 2100 K under identical tension brings about a modulus increment of 28–45 GPa. This range overlaps considerably with the tension-induced increment, indicating that both parameters play substantial and comparable roles in modulating fiber modulus under the investigated conditions. The distinct microstructural mechanisms underlying these effects are examined in detail in Section 3.2 through XRD and orientation analyses [27]. These mechanisms are temperature-driven crystallite coarsening versus tension-driven orientation enhancement.

To quantitatively characterize the saturation behavior observed in Figure 4, we fitted all modulus-versus-tension curves using an exponential saturation model: ${y=y}_{0}+A\times {[1-\exp(-x/T}_{c})]$, where y_0_ is the zero-tension modulus, A is the saturation amplitude, and Tc is the critical tension constant. The fitting curves are shown in Figure S4, and the fitting parameters are summarized in Table 1. The zero-tension modulus increases monotonically with increasing graphitization temperature: from 285.61 GPa at 1700 K to 341.86 GPa at 2100 K. This temperature-dependent saturation onset indicates that higher thermal activation energy facilitates earlier saturation of tension-induced crystallite alignment, consistent with the enhanced atomic mobility at elevated temperatures. The saturation amplitude A also increases with temperature (from 65.97 GPa at 1700 K to 114.19 GPa at 2100 K), reflecting the greater achievable modulus increment at higher graphitization temperatures. The excellent goodness-of-fit with R^2^ > 0.98 for all temperatures confirms the validity of the exponential saturation model for describing the tension–modulus relationship.

### 3.2. Microstructural Variations of Carbon Fibers

#### 3.2.1. Graphite Crystallite Size

**Figure 5:**
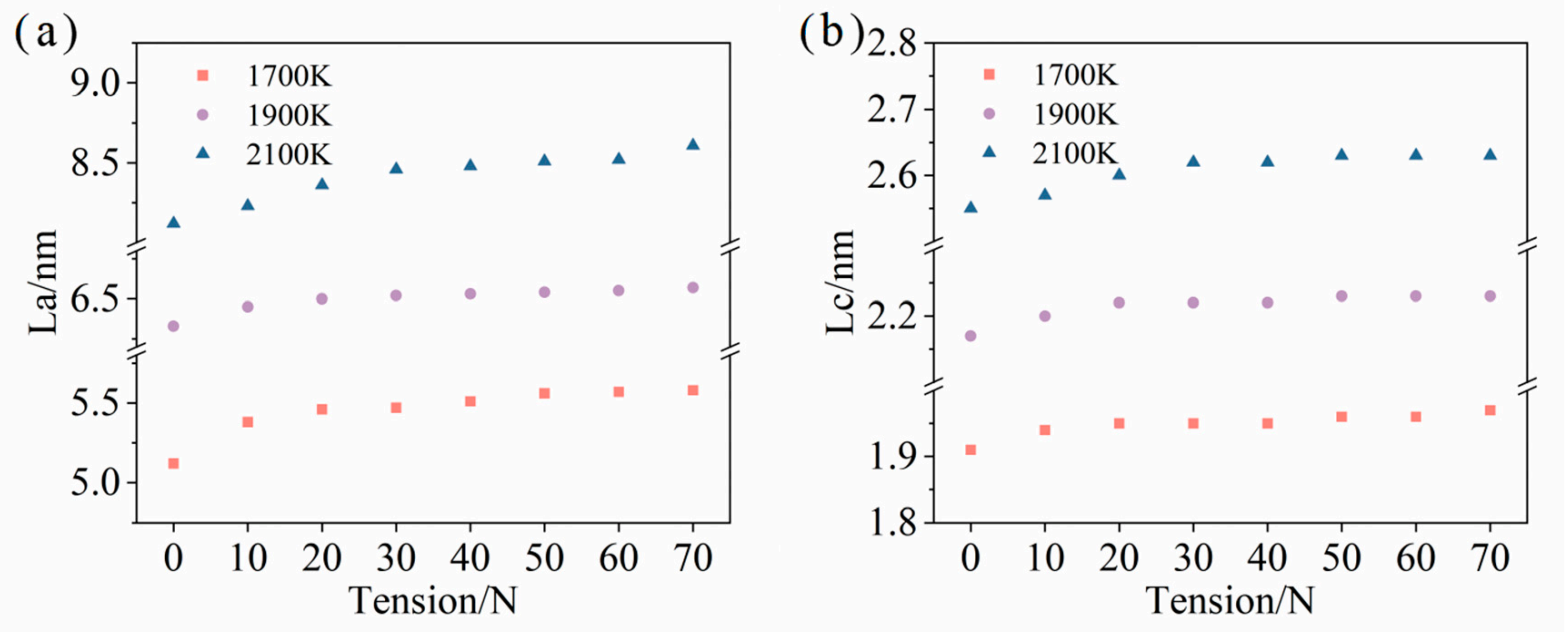
(**a**) La and (**b**) Lc of carbon fibers under different tension–temperature.

The in-plane crystallite dimension (La) and stacking height (Lc) calculated from XRD patterns (Figures S1 and S2) are plotted against applied tension under different graphitization temperatures in Figure 5. These two parameters quantitatively reflect the degree of graphitization and crystallite growth in carbon fiber bulk structure. Graphitization temperature plays a dominant role in controlling the final size of graphite crystallites. At the same tension value, both La and Lc increase significantly with rising temperature. When tension is fixed at 40 N, the La value is only 5.51 nm at 1700 K, whereas it reaches 8.48 nm at 2100 K. The same temperature-driven growth trend is observed for Lc. Higher temperature provides sufficient thermal activation energy, accelerating the rearrangement of amorphous carbon and promoting the coalescence of small crystallites into larger graphite domains [28,29]. This is consistent with the well-established graphitization law for PAN-based carbon fibers under high-temperature heat treatment. The interlayer spacing d_002_, calculated from the (002) peak position in equatorial scans, decreases with increasing graphitization temperature: from 0.345 nm at 1700 K to 0.339 nm at 2100 K (averaged across all tension levels). This reduction approaches the ideal graphite value of 0.335 nm [4], confirming the progressive graphitization of the carbon structure at elevated temperatures. The temperature dependence of d_002_ is consistent with the observed growth of Lc and La, further validating the dominant role of temperature in driving graphitization.

In sharp contrast, axial tension only induces mild changes in crystallite size within the whole loading range of 0–70 N. For the group treated at 1900 K, La only rises moderately from 6.33 nm to 6.57 nm as tension increases from 0 N to 70 N, and the corresponding variation in Lc is less than 0.06 nm. Even for the 2100 K high-temperature group, the overall fluctuation of La does not exceed 0.49 nm under continuous tension elevation. Axial tensile load mainly restricts thermal shrinkage and aligns graphite layers along the fiber axis; it cannot offer enough energy to drive grain boundary migration and crystallite coarsening [21]. As a result, tension hardly enlarges graphite crystallites, even though it greatly improves axial orientation and tensile modulus.

A small initial rise in La and Lc occurs when tension increases from 0 N to 30 N. This slight growth is attributed to the elimination of structural voids and defects under axial compression, which facilitates local ordering of carbon atoms. After tension exceeds 30 N, the crystallite size tends to plateau and remains nearly stable, with only minor random fluctuations. No obvious continuous crystallite coarsening can be detected under high tension.

The same exponential saturation analysis was applied to the crystallite size data in Figure 5a,b (Table 1), and the fitting curves are shown in Figures S5 and S6. For La, the critical tension Tc ranges from 14.36 N at 1700 K to 32.97 N at 2100 K, exhibiting a similar but less pronounced temperature dependence than that observed for modulus. The saturation amplitudes A for La are substantially smaller (0.22–0.53 nm) than those for modulus, confirming that tension primarily enhances modulus through orientation improvement rather than crystallite growth. For Lc, the Tc values range from 16.85 N to 23.10 N, with saturation amplitudes below 0.091 nm. The relatively low R^2^ values (0.9179–0.9866) for crystallite size fittings reflect the inherent scatter in XRD-derived parameters and the mild response of crystallite dimensions to tension, consistent with our conclusion that tension has only a negligible effect on crystallite coarsening.

#### 3.2.2. Microcrystalline Orientation

**Figure 6:**
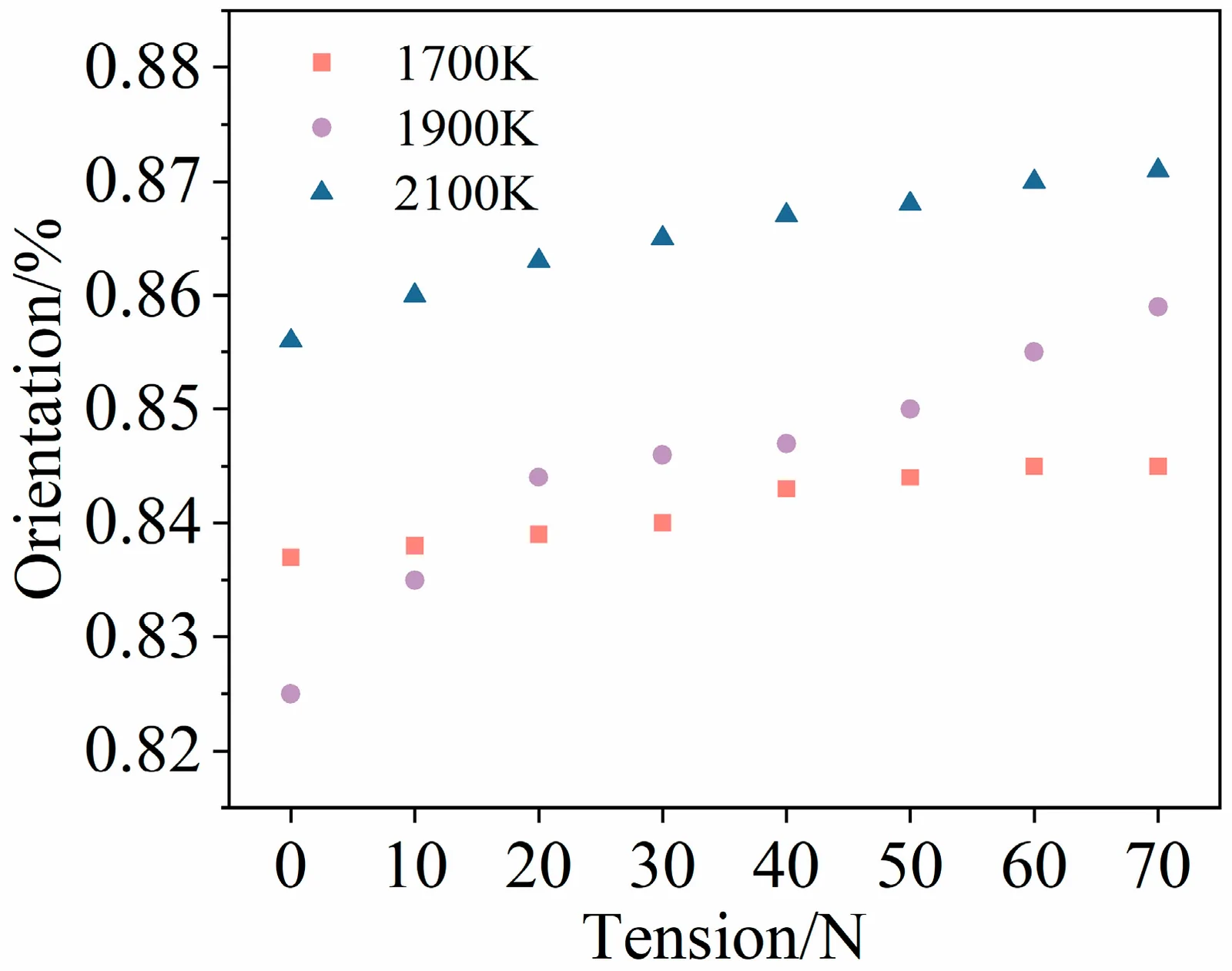
Axial orientation of carbon fibers under different tension–temperature.

The axial orientation factor calculated from azimuthal X-ray diffraction profiles (Figure S3) describes the degree to which graphite basal planes align parallel to the fiber axial direction, and this structural parameter dominates the tensile modulus of PAN-derived carbon fibers [25]. The full width at half maximum values derived from Figure S3 were used to calculate the orientation factor (π) via Equation (3), with higher π values corresponding to better axial alignment of graphite crystallites. Figure 6 summarizes the variation in orientation factor with increasing axial tension under different graphitization temperatures.

Within every fixed thermal treatment condition, the orientation factor rises steadily as tension increases from 0 N to 70 N, and no obvious plateau appears across the whole loading range. At 1900 K, the orientation factor starts from approximately 0.825 under zero tension and gradually climbs to 0.859 at the maximum tension load. Axial tension suppresses the natural thermal contraction of fiber tows during high-temperature heat treatment, and the persistent stretching force drives disordered graphite sheets to rotate toward the fiber axis, continuously improving preferred axial alignment [30]. Compared with crystallite size (La, Lc), the microcrystalline orientation is extremely sensitive to tensile load.

Graphitization temperature also exerts a positive influence on graphite alignment. Under identical tension, fibers treated at higher temperature always possess a higher orientation value. At 50 N tension, the orientation factor is 0.844 for samples processed at 1700 K and increases to 0.868 for the 2100 K group. Elevated temperature enhances the mobility of carbon atomic clusters, facilitating the reorientation of graphite lamellae under external tensile stress. The effect of tension on orientation, however, exhibits a clear temperature dependence. Raising tension from 0 N to 70 N increases the orientation factor by approximately 0.034 at 1900 K, whereas the same tension increment yields only 0.008 at 1700 K and 0.015 at 2100 K. This temperature-dependent efficiency suggests that the interplay between thermal mobility and mechanical constraint is most favorable at intermediate temperatures: at 1700 K, limited thermal mobility restricts lamellar rotation even under tension; at 2100 K, extensive crystallite growth and alignment have already occurred during the high-temperature treatment, leaving less room for further tension-induced improvement [27,30]. The effect of tension on orientation becomes comparable to that of temperature variation only up to a tension of approximately 40 N; beyond this threshold, the tension effect continues to increase at 1900 K, while the temperature effect saturates earlier. This indicates an interactive, rather than simply additive, relationship between temperature and tension in controlling orientation.

The orientation factor data in Figure 6 were also fitted using the exponential saturation model (Table 1), and the fitting curves are shown in Figure S7. The critical tension Tc increases from 18.85 N at 1700 K to 46.98 N at 2100 K, closely following the temperature dependence observed for modulus. This parallel behavior confirms that the saturation of modulus enhancement is governed by the saturation of microcrystalline orientation. The saturation amplitudes A for orientation range from 0.019 to 0.037, corresponding to relative improvements over the zero-tension baseline. The high R^2^ values (>0.9606) demonstrate that the exponential model effectively captures the orientation–tension relationship across all temperatures.

**Figure 7:**
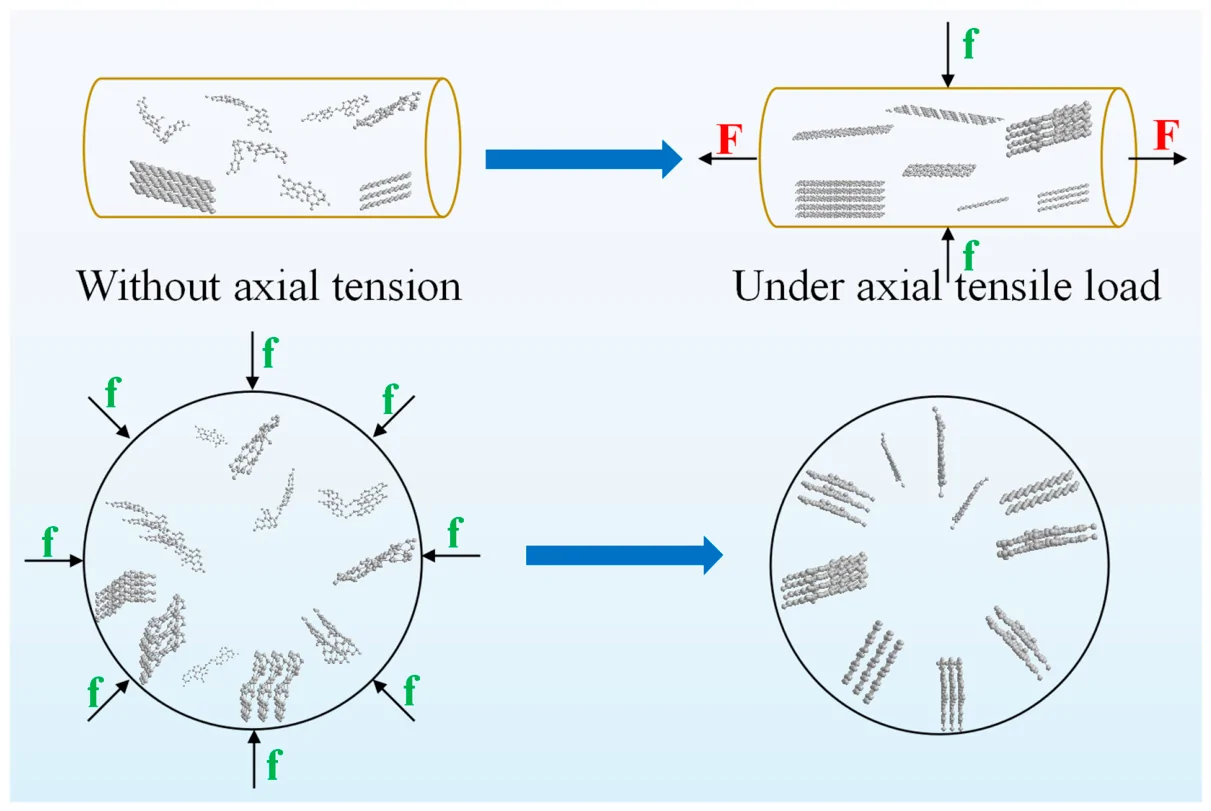
Schematic diagram of microcrystalline orientation carbon fibers under axial tension.

Figure 7 presents a schematic illustration of internal graphite stacking evolution in PAN-based carbon fibers subjected to axial tension during high-temperature graphitization. Without applied tension, graphitic domains are randomly distributed throughout the filament, with lamellae adopting diverse angular orientations relative to the fiber axis. When sustained axial tension F is imposed, thermally activated fiber shrinkage generates uniform radial compressive stress f across the cross-section. This radial stress acts as the key driving force for graphite lamella rearrangement. Under the combined thermal and mechanical fields, graphitic fragments rotate toward the radial direction of the fiber cross-section.

Such structural transformation yields two competing effects that jointly govern Sac content. On one hand, tensile constraint promotes moderate crystallite growth, which reduces the number of edge carbon sites within graphite domains. On the other hand, radial reorientation pushes more lamellar boundaries toward the fiber outer surface, exposing additional unsaturated edge carbon atoms [25]. These counteracting structural changes explain the experimental observation that Sac remains nearly constant across a broad tension range at fixed graphitization temperature. Although axial tension effectively improves the preferred orientation of graphitic crystallites and enhances tensile modulus, it does not continuously elevate or deplete Sac concentration.

**Figure 8:**
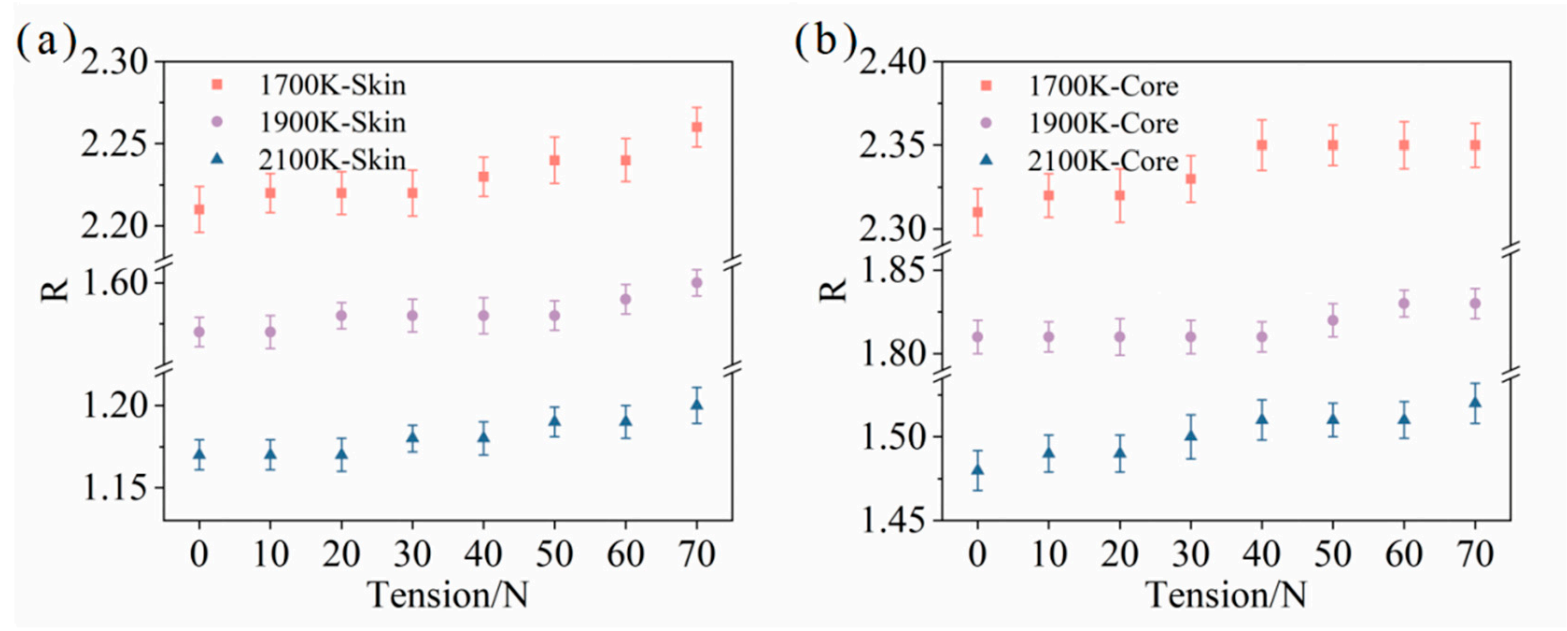
Evolution of R value for HMCFs under different tension–temperature: (**a**) R values of the fiber skin region; (**b**) R values of the fiber core region.

Figures S8–S10 show the Raman spectra of skin and core for HMCFs treated at different temperature under various tension. The R value (I_D_/I_G_) serves as a direct indicator of local graphitization level. Figure 8 presents the evolution of R value under different tensions and different temperatures.

At each fixed heat treatment temperature, the R-skin value only fluctuates mildly when axial tension increases continuously from 0 N to 70 N, without showing any obvious rising or falling trend. At 1700 K, the R-skin value remains stable between 2.21 and 2.26 throughout the whole tension gradient, and no systematic decline caused by crystallite growth can be observed. As discussed in the previous XRD analysis, tension only induces slight coarsening of graphite crystallites. This minor grain growth would theoretically reduce the number of boundary carbon atoms and lower the R value. However, axial tension produces radial compressive stress during fiber shrinkage, which forces graphite basal planes to rotate toward the fiber radial direction and expose more edge sites on the outer surface. These two competing structural changes counteract each other, so the surface defect density stays nearly constant regardless of tensile load.

Graphitization temperature still dominates the final R-skin value. Under identical tension, fibers treated at lower temperature always deliver a much higher R value. When tension is fixed at 40 N, the R-skin reaches 2.23 at 1700 K, whereas it falls to only 1.18 for the 2100 K group. High-temperature graphitization drastically accelerates the growth of graphite crystallites and eliminates boundary defects, which makes the fiber surface chemically inert and weakens interfacial bonding with epoxy resin [31]. Unlike tension, elevated temperature cannot bring about radial lamella rearrangement to compensate for the loss of edge carbon sites. This explains why the surface polarity inevitably declines if we only rely on high-temperature treatment to improve fiber modulus.

It is worth noting that the skin-core structural difference does not expand or shrink significantly under varying tension. Axial tensile stress acts uniformly along the fiber axial direction and only causes overall radial compression. It cannot alter the radial temperature gradient inside the fiber furnace zone or change the difference in graphitization degree between the fiber skin and core regions [32]. As a result, tension will not aggravate the radial structural heterogeneity, which is another advantage of the tension–temperature synergistic processing route.

### 3.3. Temperature-Dominated Surface Polarity and Interfacial Properties

**Figure 9:**
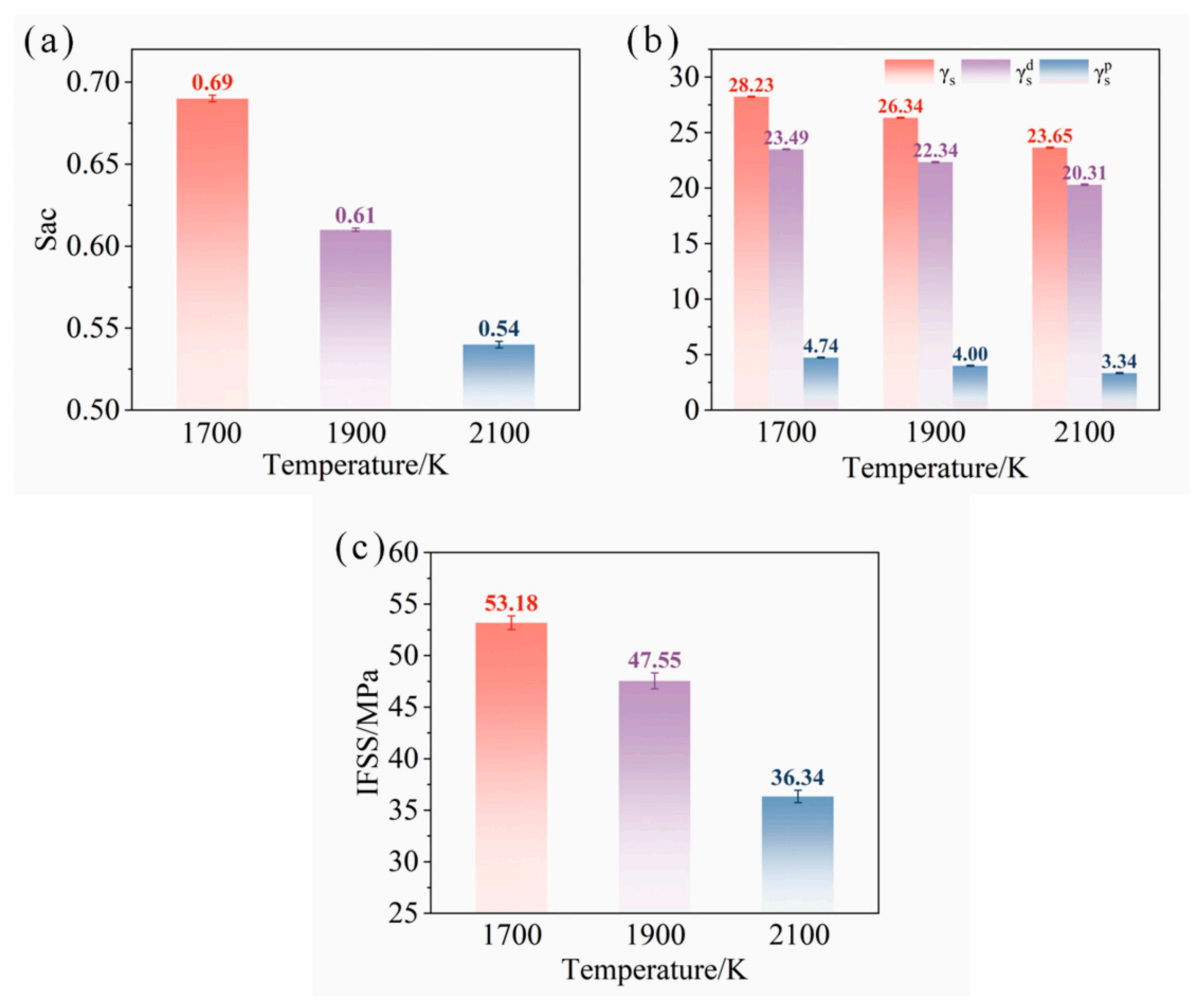
Surface polarity and interfacial properties as a function of graphitization temperature: (**a**) Sac; (**b**) surface energy component ($\gamma_{s}$, $\gamma_{s}^{d}$, $\gamma_{s}^{p}$); (**c**) IFSS.

Figure 9 presents the surface polarity and interfacial properties of HMCFs as a function of graphitization temperature, with each data point averaged across all tension levels (0–70 N). This representation is justified by the tension independence of these parameters, as confirmed by linear fits of the individual tension-dependent curves, yielding slopes approximately equal to zero (Figure S11). This tension-independence results from the offsetting effects of two concurrent structural changes: mild crystallite coarsening and radial lamella exposure. Their competition keeps the surface-active carbon content stable under varying tensile load [25,33].

Figure 9a shows the evolution of the Sac parameter with graphitization temperature. The Sac value decreases markedly from 0.692 ± 0.002 at 1700 K to 0.542 ± 0.002 at 2100 K with a reduction of approximately 22%. This temperature-driven decline reflects the accelerated coalescence of graphite crystallites at elevated temperatures, which eliminates edge-active carbon sites on the fiber surface [34]. The strong negative temperature dependence (linear regression slope: −1.50 × 10^−4^ K^−1^, R^2^ = 0.999) confirms that temperature is the decisive factor governing the intrinsic surface polarity of HMCFs.

Figure 9b presents the surface energy components as functions of temperature. The total surface energy ($\gamma_{s}$) decreases from 28.23 ± 0.05 mN·m^−1^ at 1700 K to 23.65 ± 0.05 mN·m^−1^ at 2100 K. This decrease is primarily attributed to the dispersive component ($\gamma_{s}^{d}$), which drops from 23.49 ± 0.04 mN·m^−1^ to 20.31 ± 0.06 mN·m^−1^ over the same temperature range, while the polar component ($\gamma_{s}^{p}$) exhibits a more modest decline from 4.74 ± 0.05 mN·m^−1^ to 3.34 ± 0.05 mN·m^−1^. Well-stacked sp^2^ carbon lattices give rise to stable non-polar dispersion forces that show sensitivity to processing temperature. Their abundance is directly reflected by the Sac parameter. Unsaturated sp^2^ edge carbon sites and residual heteroatom groups on the fiber surface serve as polar interaction sites for epoxy matrices [35].

Figure 9c illustrates the IFSS values averaged across all tension levels at each temperature. IFSS decreases from 53.18 ± 0.66 MPa at 1700 K to 36.34 ± 0.60 MPa at 2100 K, following a linear trend with temperature (slope: −0.028 MPa·K^−1^, R^2^ = 0.999). The strong positive correlation between IFSS and Sac confirms that the surface-active carbon content is the primary determinant of fiber–matrix adhesion [31].

Collectively, these results demonstrate that graphitization temperature plays the dominant role in regulating both the surface polarity and interfacial bonding strength of HMCFs, while tension effectively enhances bulk modulus without sacrificing surface polarity.

### 3.4. Response Surface Analysis of Coupled Processing, Surface and Interfacial Performance Parameters

**Figure 10:**
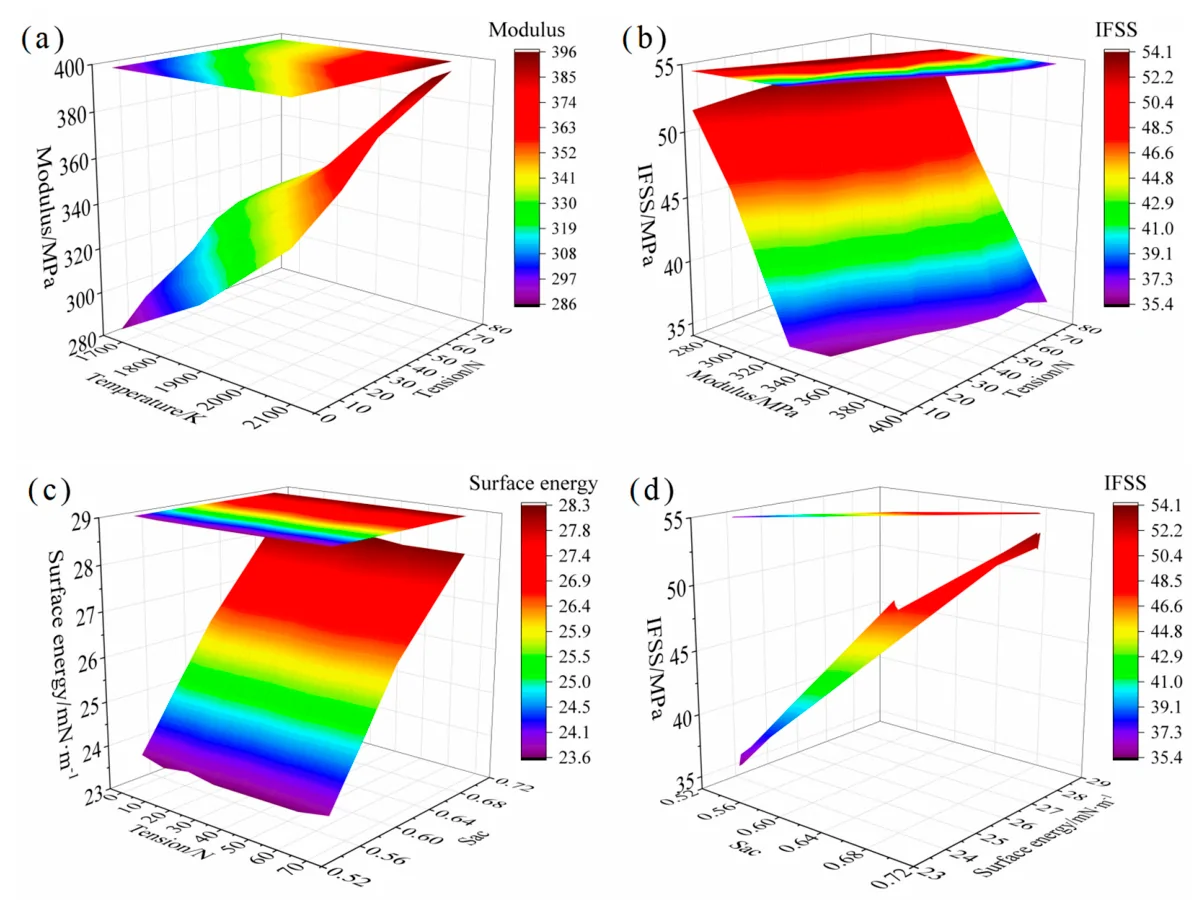
Three-dimensional response surfaces: (**a**) tensile modulus as functions of temperature and tension; (**b**) IFSS as functions of modulus and tension; (**c**) surface energy as functions of tension and Sac; (**d**) IFSS as functions of Sac and surface energy.

Figure 10 presents four three-dimensional response surfaces constructed to decouple the independent and synergistic effects of temperature and tension on modulus, IFSS, and surface energy. The fitted quadratic regression models all yield adjusted determination coefficients above 0.93, confirming reliable predictive capability.

Figure 10a shows the 3D surface for tensile modulus, which exhibits a consistent upward incline along both temperature and tension axes. The surface gradient along the tension coordinate is steeper, confirming that axial hot-stretching contributes more substantially to modulus elevation. Most notably, multiple coordinate points on the same modulus isoline indicate that different temperature–tension combinations can yield identical modulus values, enabling modulus preservation while tuning surface polarity.

Figure 10b shows the IFSS response surface plotted against modulus and tension. IFSS changes drastically along the modulus axis but remains constant across the tension range at any fixed modulus, confirming that tension does not affect interfacial performance. Fibers with identical modulus produced via low-temperature high-tension processing exhibit significantly higher IFSS than their high-temperature low-tension counterparts.

Figure 10c presents the surface energy response as a function of tension and Sac. Total surface energy increases monotonically with Sac but shows no systematic shift with tension at a fixed Sac, confirming that tension does not alter the intrinsic Sac-surface–energy relationship.

Figure 10d shows the IFSS response as a function of Sac and surface energy. IFSS rises continuously with both parameters in a near-linear manner, indicating additive effects without mutual interference. Combined with the dual-factor processing surfaces, these correlations establish a complete logic chain: temperature dominates Sac and surface energy, tension independently controls axial orientation and modulus, and IFSS is determined by surface polarity rather than bulk mechanical properties.

### 3.5. Tension–Temperature Coupling Strategy for High-Modulus High-Polarity Carbon Fibers

Table 2 compares two processing routes that yield equivalent modulus (~350 GPa): high-temperature low-tension (2100 K, 10 N) and low-temperature high-tension (1900 K, 60 N). The high-temperature route produces larger crystallites (La = 8.23 nm, Lc = 2.57 nm) with lower surface polarity (Sac = 0.539), while the low-temperature high-tension route retains finer crystallites (La = 6.55 nm, Lc = 2.23 nm) with significantly higher Sac (0.612) and IFSS (48.8 MPa vs. 35.4 MPa). The nearly identical moduli (351 GPa vs. 350 GPa) confirm that tension fully offsets the modulus loss from reduced temperature through enhanced axial orientation.

**Figure 11:**
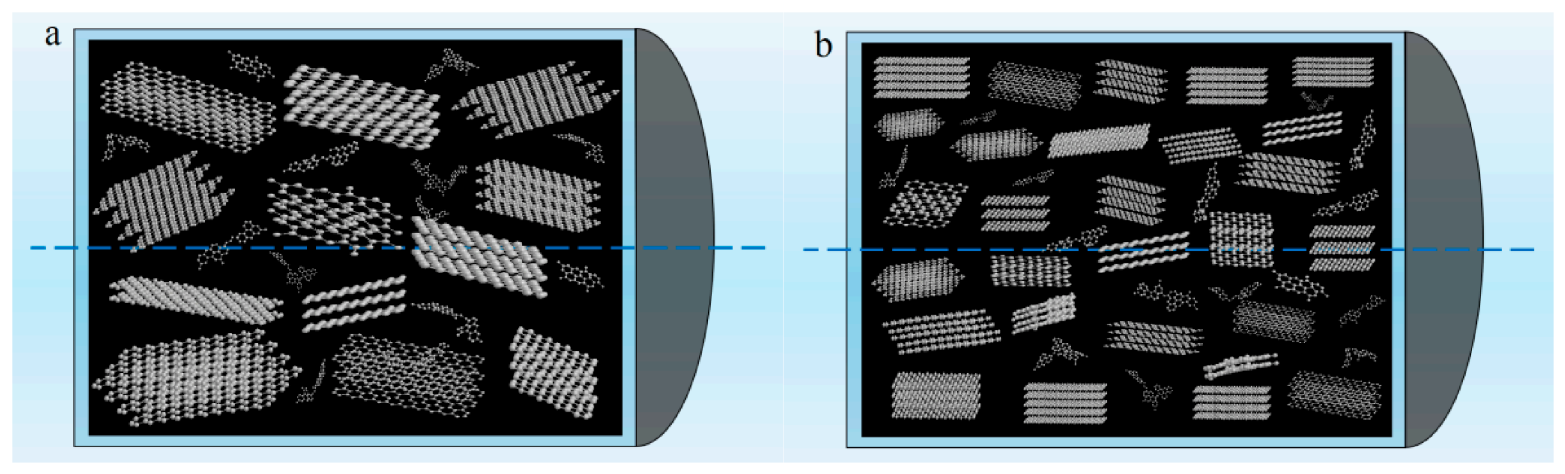
Schematic diagram of carbon fiber structure prepared with different temperature–tension combinations: (**a**) high-temperature low-tension combination; (**b**) low-temperature high-tension combination.

Figure 11 schematically contrasts the two microstructural states. High-temperature low-tension processing (Figure 11a) yields large, thick-stacked crystallites with random orientation and scarce edge sites. Low-temperature high-tension processing (Figure 11b) produces smaller crystallites with abundant edge carbon defects and superior axial alignment. The synergy of fine crystallites that retain surface-active sites and high tension that promotes axial orientation enables equivalent modulus with enhanced interfacial performance, thereby overcoming the conventional modulus–polarity trade-off.

## 4. Conclusions

This paper systematically decouples the regulatory effects of graphitization temperature and axial tension on the microstructure, surface polarity, and interfacial bonding of PAN-based HMCFs. Combined XRD, Raman, contact angle and microdroplet debond characterizations of samples prepared at 1700–2100 K under 0–70 N tension confirm that temperature governs graphite crystallite growth and surface-active carbon content, while tension primarily enhances axial lamellar orientation with negligible influence on crystallite dimensions. A competitive balance between mild crystallite coarsening and radial graphite lamella exposure induced by tensile shrinkage keeps the Sac value, surface energy, and IFSS nearly unchanged across the entire tension range. The Sac parameter exhibits strong linear correlations with total surface energy (R^2^ > 0.95) and IFSS (R^2^ > 0.93), validating its reliability as a quantitative polarity indicator.

Based on the experimental results, the recommended processing condition for achieving balanced modulus and enhanced interfacial performance is 1900 K graphitization temperature combined with 60 N axial tension. This combination yields a tensile modulus of ~350 GPa, a Sac of 0.612, and an IFSS of 48.8 MPa. For applications requiring different modulus targets, the response surface analysis indicates that a processing window of 1850–1950 K with 50–70 N tension can achieve comparable modulus while maintaining elevated surface polarity. Comparative analysis of two representative routes confirms that this low-temperature high-tension synergistic route fully compensates for modulus loss from moderate temperature reduction through enhanced axial orientation, achieving a 13.5% higher Sac and a 37.9% improvement in IFSS relative to the conventional high-temperature low-tension scheme. This one-step in situ processing strategy eliminates the need for ultrahigh-temperature graphitization and additional post-surface modification, offering a scalable industrial pathway for producing HMCFs with superior intrinsic fiber–matrix interfacial performance.

## Figures and Tables

**Table 1:** Exponential saturation fitting parameters for modulus, crystallite size, and orientation as functions of tension at different graphitization temperatures.

Parameter	Temperature/K	y_0_/GPa	A	Tc/N	R^2^
Modulus/GPa	1700	285.61	65.97	55.40	0.9872
	1900	308.31	70.04	71.95	0.9941
	2100	341.86	114.19	98.31	0.9859
La/nm	1700	5.13	0.22	14.36	0.9743
	1900	6.33	0.43	14.79	0.9866
	2100	8.11	0.53	32.97	0.9784
Lc/nm	1700	1.91	0.050	16.85	0.9179
	1900	2.17	0.059	18.66	0.9757
	2100	2.55	0.091	23.10	0.9758
Orientation/%	1700	0.837	0.029	18.85	0.9606
	1900	0.826	0.037	38.68	0.9628
	2100	0.856	0.019	46.98	0.9971

Note: y_0_ is the initial value at zero tension; A is the saturation amplitude; Tc is the critical tension constant; R^2^ is the coefficient of determination.

**Table 2:** Sample parameters with modulus approximately 350 GPa.

Temperature /K	Tension /N	Modulus /GPa	La /nm	Lc /nm	Orientation	Sac	Surface Energy /mN·m^−1^	IFSS /MPa
2100	10	351	8.23	2.57	0.860	0.539	23.6	35.4
1900	60	350	6.55	2.23	0.855	0.612	26.3	48.8

## Data Availability

The original contributions presented in this study are included in the article/Supplementary Material. Further inquiries can be directed to the corresponding authors.

## Supplementary Materials

The following supporting information can be downloaded at: https://www.mdpi.com/article/10.3390/ma19163514/s1, Table S1: The physical properties of HMCFs with different temperatures and tensions; Table S2: Thermodynamic parameters of test liquids at 25 °C; Figure S1: The equatorial XRD patterns of HMCFs treated at various tension with different temperature (1700 K, 1900 K, 2100 K), The equatorial scan for d002 and Lc from the (002) peak; Figure S2: The meridional XRD patterns of HMCFs treated at various tension with different temperature (1700 K, 1900 K, 2100 K), the meridional scan for La from the (100) peak; Figure S3: The azimuthal XRD patterns of HMCFs treated at various tension with different temperature (1700 K, 1900 K, 2100 K), The azimuthal scan for orientation factor π from the (002) azimuthal intensity distribution; Figure S4: Exponential saturation fitting of tensile modulus as functions of applied tension at three graphitization temperatures (1700 K, 1900 K, and 2100 K); Figure S5: Exponential saturation fitting of La as functions of applied tension at three graphitization temperatures (1700 K, 1900 K, and 2100 K); Figure S6: Exponential saturation fitting of Lc as functions of applied tension at three graphitization temperatures (1700 K, 1900 K, and 2100 K); Figure S7: Exponential saturation fitting of axial orientation as functions of applied tension at three graphitization temperatures (1700 K, 1900 K, and 2100 K); Figure S8: The Raman spectra of skin and core for HMCFs treated at 1700 K under various tension; Figure S9: The Raman spectra of skin and core for HMCFs treated at 1900 K under various tension; Figure S10: The Raman spectra of skin and core for HMCFs treated at 2100 K under various tension; Figure S11: Variations of (a) Sac, (b) total surface energy-$\gamma_{s}$, (c) polar component-$\gamma_{s}^{p}$, (d) dispersive component-$\gamma_{s}^{d}$, and (e) IFSS as a function of tension for carbon fibers graphitized at 1700 K, 1900 K and 2100 K.
