# Supplementary material for: Tension–Temperature Synergy in Tailoring Surface Polarity and Interfacial Properties of High-Modulus PAN-Based Carbon Fibers
Source: Materials (Basel). 2026 Aug 19;19(16):3514. doi: 10.3390/ma19163514 (PMC13514715; doi:10.3390/ma19163514)
Supplement: Supplementary file 1 [file materials-19-03514-s001.zip › materials-4473436-supplementary.pdf]

Tension-Temperature Synergy in Tailoring Surface Polarity and Interfacial properties of High-Modulus PAN-Based Carbon Fibers

Aijun Gao*[1], Tiansheng Fan[1]， Weize Tian[1]， Panpan Xu[2], Hailong Zhang*[3]

1. College of Materials Science and Engineering, Beijing University of Chemical Technology, Beijing 100029, China

2. Jiangsu Hengshen Co.,Ltd, Jiangsu Danyang, 212300, China

3. School of Materials Science and Engineering, North China University of Water Resources and Electric Power, Zhengzhou, 450045, China

Corresponding authors:

1. Aijun Gao; E-mail: gaoaj@buct.edu.cn; Telephone: 13401193894

2. Hailong Zhang; E-mail: zhanghailongql@163.com; Telephone: 13838161314.

Table S1. The physical properties of HMCFs with different temperatures and tensions.

Temperature/K	Tension/N	Modulus/GPa	Density/(g/cm^3)	Linear Density/(g/m)
1700K	0	286±3.2	1.797±0.012	0.498±0.015
	10	297±3.1	1.799±0.013	0.494±0.016
	20	304±4.0	1.799±0.011	0.485±0.014
	30	311±2.8	1.800±0.012	0.479±0.015
	40	322±4.2	1.798±0.007	0.474±0.013
	50	327±2.5	1.798±0.009	0.473±0.015
	60	329±2.8	1.800±0.013	0.472±0.016
	70	330±4.2	1.796±0.008	0.471±0.013
1900K	0	309±3.9	1.784±0.012	0.489±0.019
	10	320±5.2	1.788±0.014	0.479±0.017
	20	331±4.7	1.785±0.014	0.477±0.018
	30	338±3.5	1.787±0.011	0.475±0.016
	40	346±3.9	1.791±0.015	0.476±0.016
	50	349±3.5	1.788±0.009	0.476±0.015
	60	350±4.0	1.788±0.013	0.472±0.017
	70	352±3.4	1.789±0.010	0.472±0.018
2100K	0	343±2.7	1.795±0.016	0.481±0.016
	10	351±4.1	1.793±0.013	0.481±0.017
	20	359±4.5	1.796±0.015	0.483±0.019
	30	370±4.3	1.795±0.016	0.473±0.015
	40	381±2.9	1.797±0.014	0.468±0.016
	50	391±3.5	1.798±0.012	0.467±0.017
	60	395±4.9	1.798±0.013	0.465±0.018
	70	396±4.5	1.798±0.012	0.463±0.016

Table S2. Thermodynamic parameters of test liquids at 25°C.

Liquid	γ_L (mN/m)	γ_L^d (mN/m)	γ_L^p (mN/m)
Water	72.8	29.1	43.7
Diiodomethane	50.8	50.4	0.4

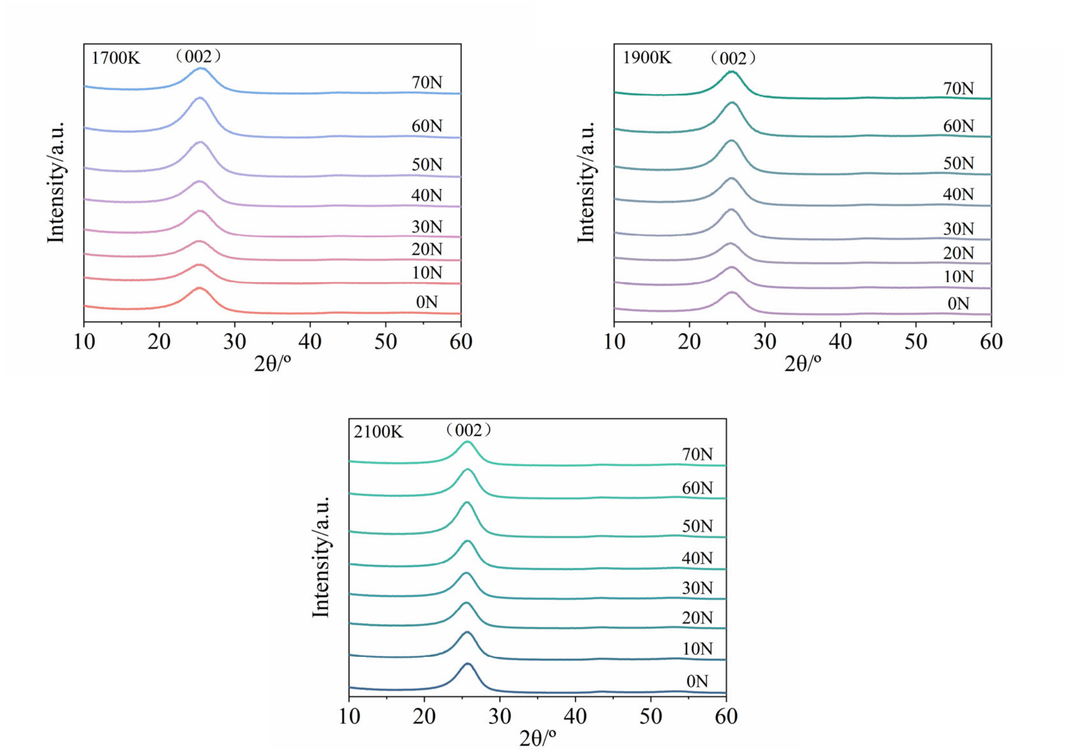

Figure S1. The equatorial XRD patterns of HMCFs treated at various tension with different temperature (1700K, 1900K, 2100K).

(The equatorial scan for d_{002} and Lc from the (002) peak)

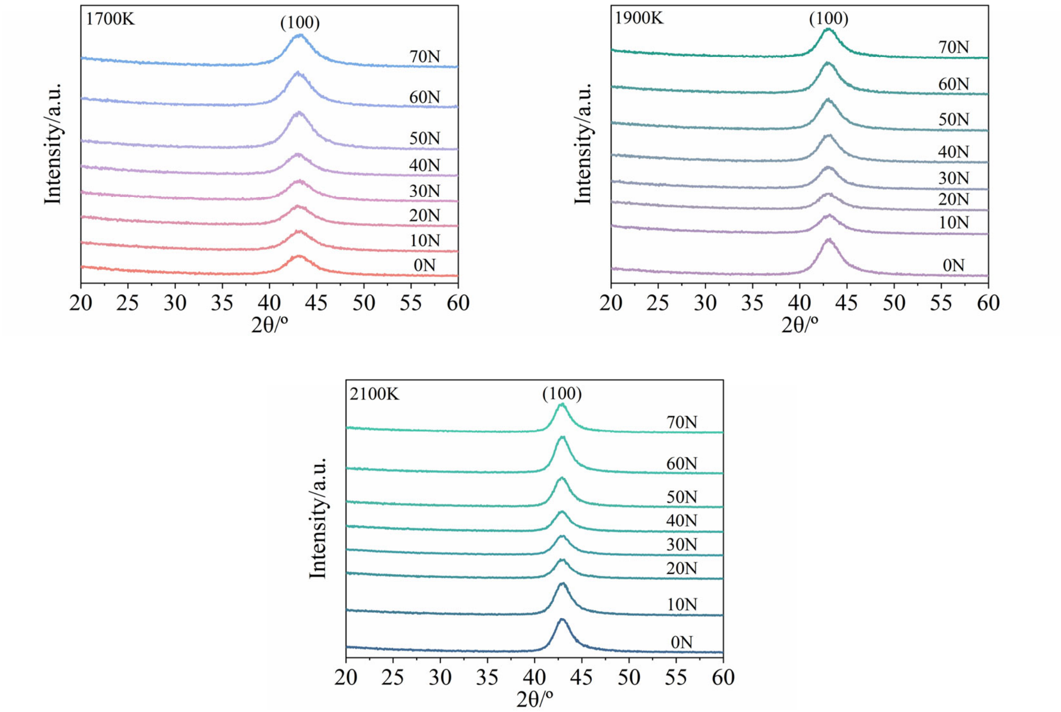

Figure S2. The meridional XRD patterns of HMCFs treated at various tension with different temperature (1700K, 1900K, 2100K).

(The meridional scan for La from the (100) peak)

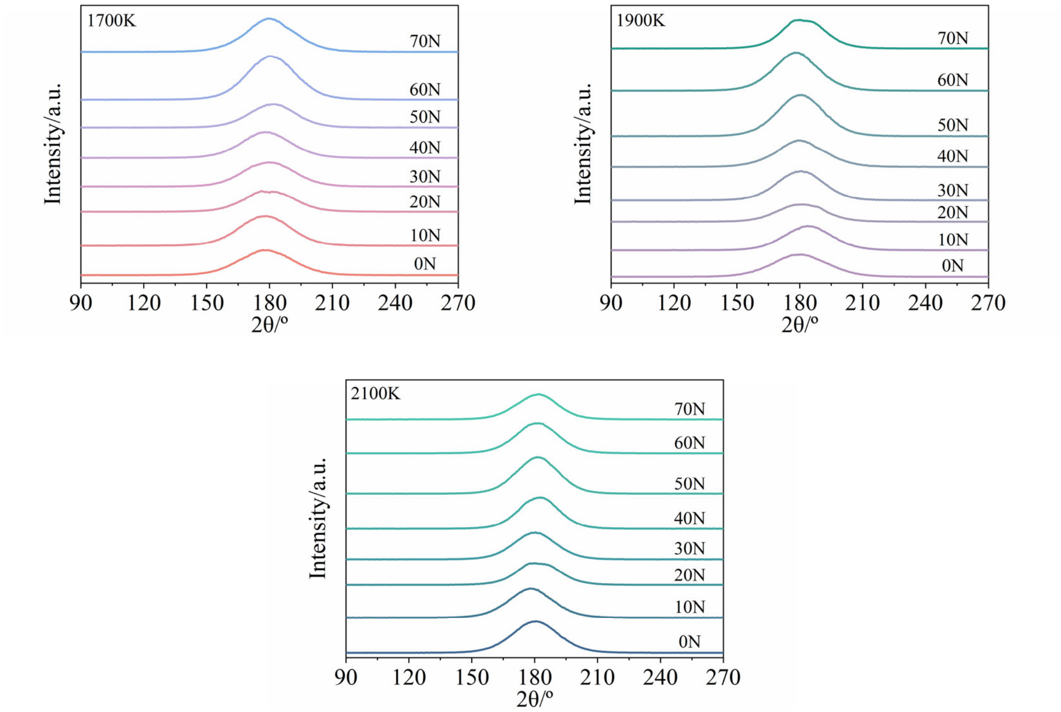

Figure S3. The azimuthal XRD patterns of HMCFs treated at various tension with different temperature (1700K, 1900K, 2100K).

(The azimuthal scan for orientation factor π from the (002) azimuthal intensity distribution)

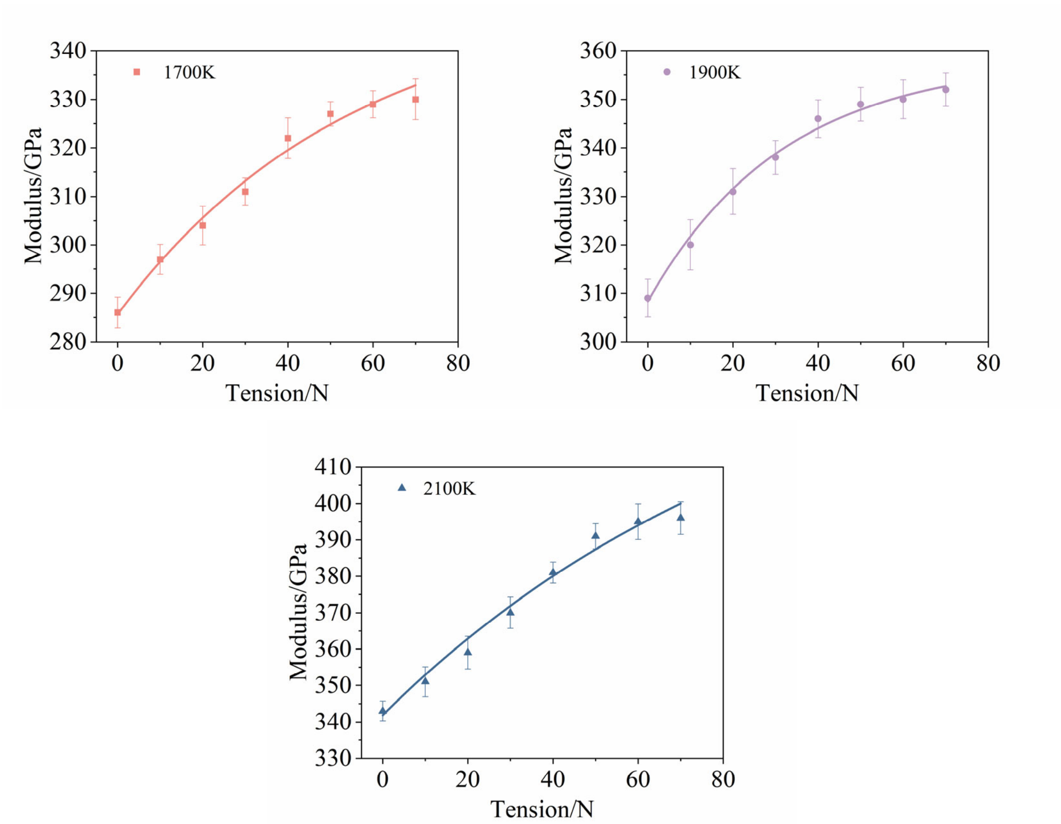

Figure S4. Exponential saturation fitting of tensile modulus as functions of applied tension at three graphitization temperatures (1700 K, 1900 K, and 2100 K).

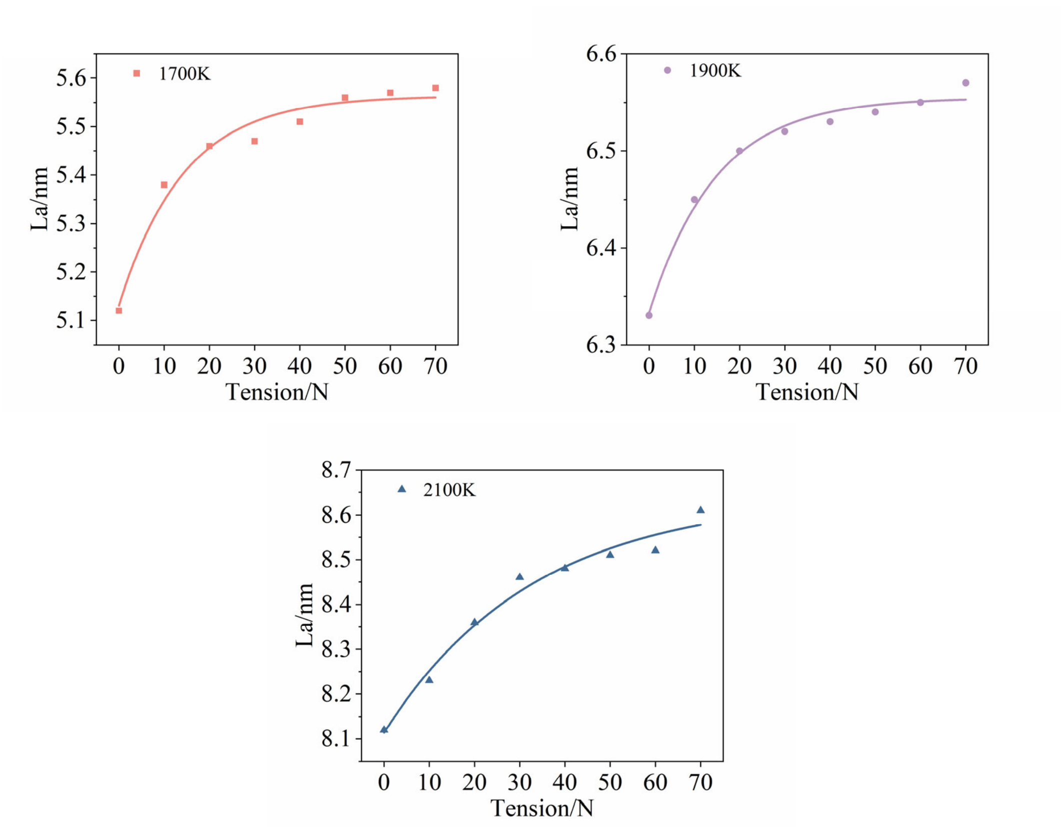

Figure S5. Exponential saturation fitting of La as functions of applied tension at three graphitization temperatures (1700 K, 1900 K, and 2100 K).

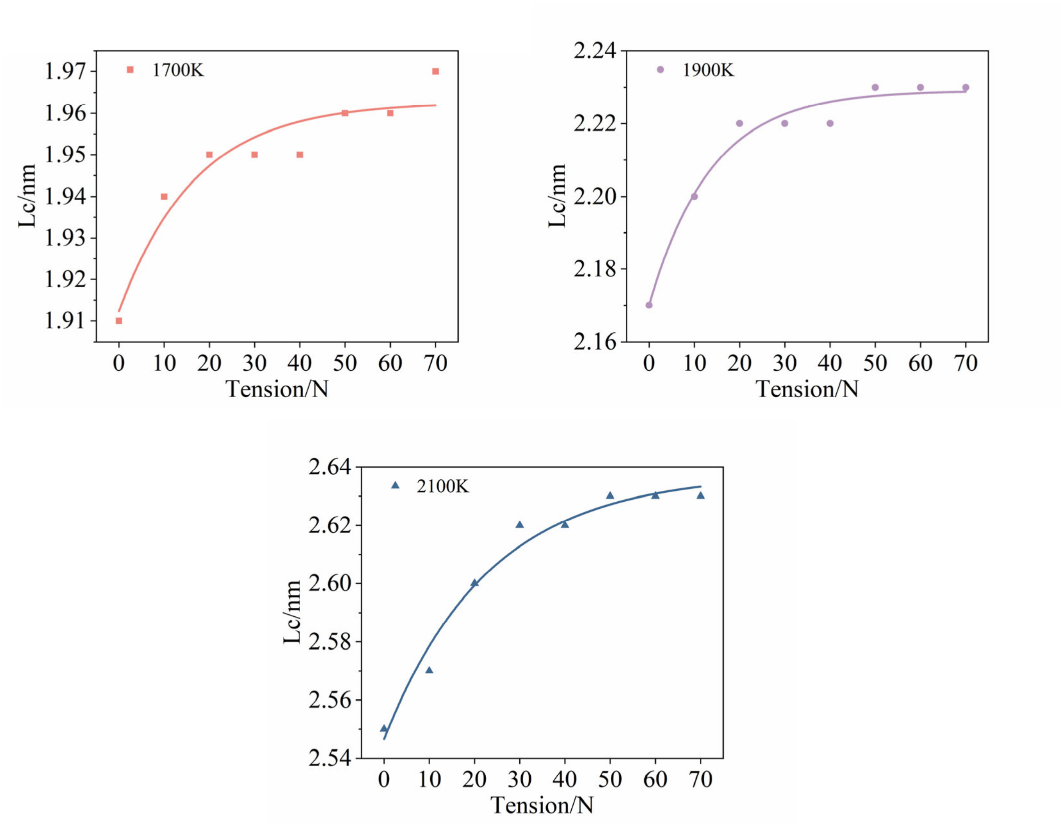

Figure S6. Exponential saturation fitting of Lc as functions of applied tension at three graphitization temperatures (1700 K, 1900 K, and 2100 K).

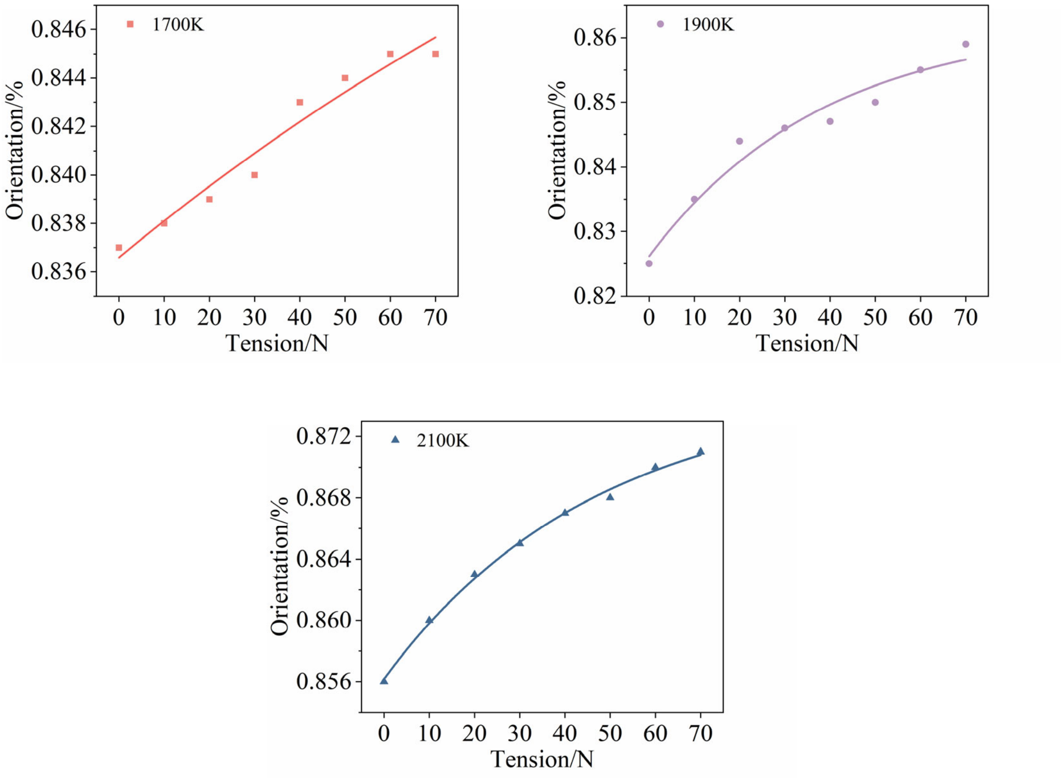

Figure S7. Exponential saturation fitting of axial orientation as functions of applied tension at three graphitization temperatures (1700 K, 1900 K, and 2100 K).

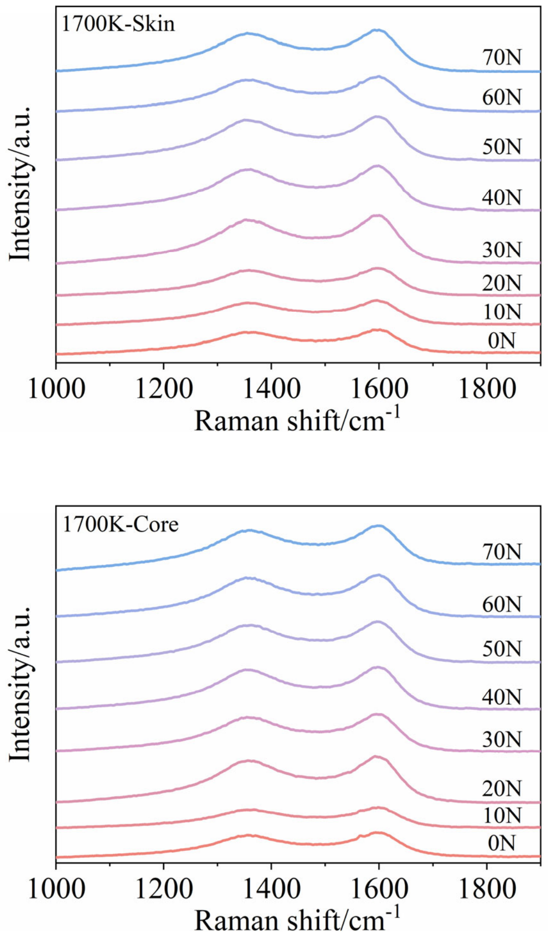

Figure S8. The Raman spectra of skin and core for HMCFs treated at 1700K under various tension.

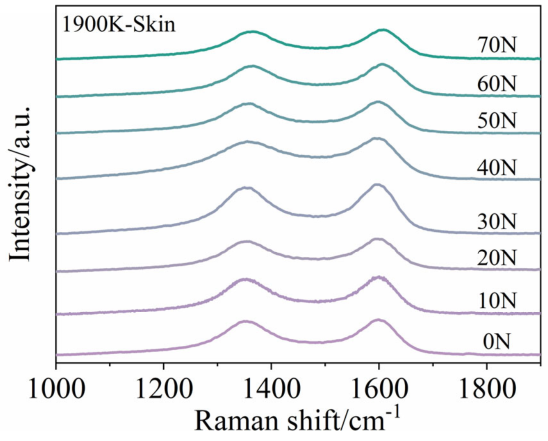

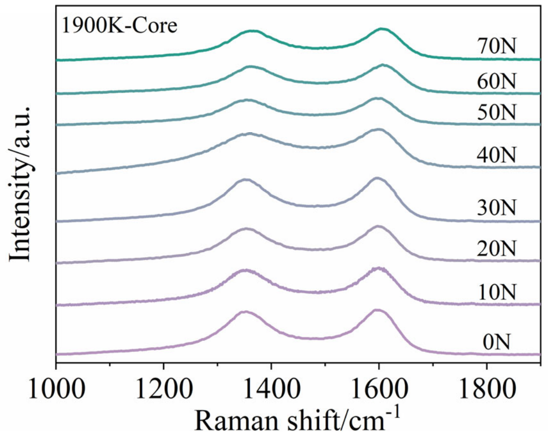

Figure S9. The Raman spectra of skin and core for HMCFs treated at 1900K under various tension.

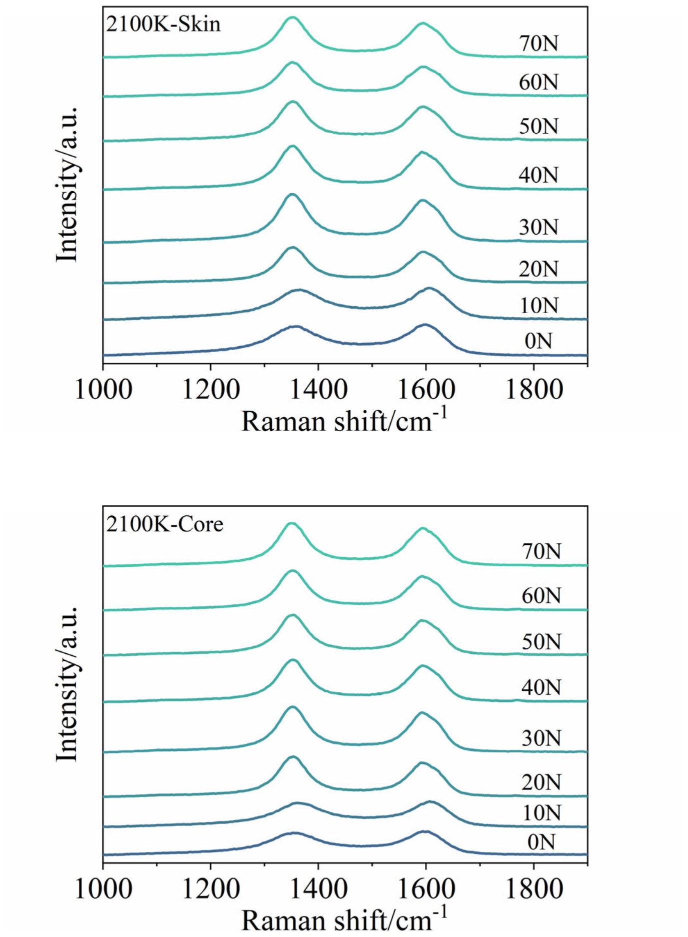

Figure S10. The Raman spectra of skin and core for HMCFs treated at 2100K under various tension.

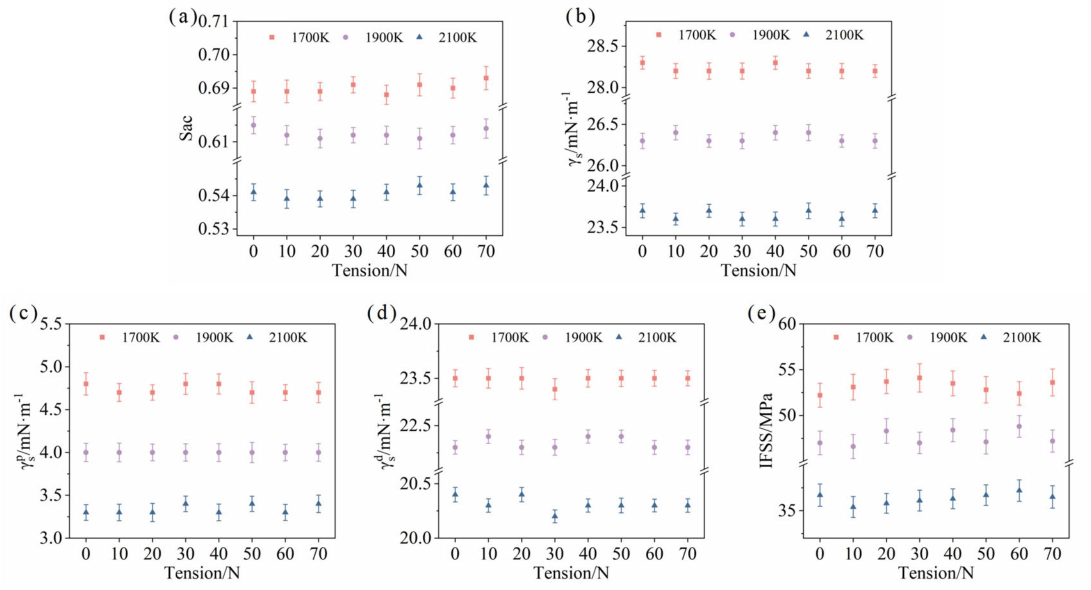

Figure S11. Variations of (a) Sac, (b) total surface energy-γ_s, (c) polar component-γ_s^p, (d) dispersive component-γ_s^d, and (e) IFSS as a function of tension for carbon fibers graphitized at 1700 K, 1900 K and 2100 K.
